# Use of a risk characterisation approach to contextualise the safety profile of new rheumatoid arthritis treatments: a case study using tofacitinib

**DOI:** 10.1007/s10067-016-3359-x

**Published:** 2016-07-28

**Authors:** Jeffrey R. Curtis, Richard Zhang, Sriram Krishnaswami, Andrew Anisfeld, Yan Chen, Sander Strengholt, Connie Chen, Jamie Geier

**Affiliations:** 1 The University of Alabama at Birmingham, 1720 2nd Ave S, Birmingham, AL 35233 USA; 2 Pfizer Inc, 235 East 42nd Street, New York, NY 10017 USA; 3 Pfizer Inc, Eastern Point Road, Groton, CT 06340 USA; 4 Pfizer Inc, 500 Arcola Rd, Collegeville, PA 19426 USA; 5 Pfizer Inc, Rivium Westlaan 142, 2909 LD Capelle a/d IJssel, The Netherlands

**Keywords:** Exposure, Rheumatoid arthritis, Safety, Tofacitinib, Tumour necrosis factor inhibitors

## Abstract

Tofacitinib is an oral Janus kinase inhibitor for the treatment of rheumatoid arthritis (RA). To characterise the relative safety profile of tofacitinib to biologic disease-modifying antirheumatic drugs (bDMARDs), the accrued patient-years (pt-yrs) of exposure needed in an RA clinical trial programme to detect a potential increase in risk of specific adverse events (AEs) was determined. This case study/framework was constructed on the pt-yrs’ accrual within pooled phase (P)1, P2 and P3, as well as long-term extension, studies of tofacitinib in RA (March 2015 data-cut) and published AE incidence rates for bDMARDs. Sample size calculations were based on a Poisson distribution to estimate pt-yrs’ exposure required for 90 % probability that the lower bound of the 95 % confidence interval for tofacitinib/bDMARD would be >1, assuming that tofacitinib rates were 1.2×/1.5×/2.0× greater than comparator rates. AE rates for bDMARDs were derived from sources intended to optimise similarity with the tofacitinib database in terms of baseline characteristics, study duration and follow-up. Based on the tofacitinib exposure accrued (19,406 pt-yrs), data were sufficient (90 % probability) to detect potential differences over external bDMARD comparator rates in serious infections (≥1.2×), malignancies (excluding non-melanoma skin cancer [NMSC]), NMSC, major adverse cardiovascular events (MACE) and lymphoma (each ≥1.5×), as well as opportunistic infections and gastrointestinal perforations (≥2×), should they exist. This risk characterisation approach can support the comparative safety of new RA medications. To date, tofacitinib safety appears similar to approved published data from bDMARDs with respect to serious infections, malignancies (excluding NMSC), NMSC, MACE, lymphoma, opportunistic infections and gastrointestinal perforations.

**Electronic supplementary material:**

The online version of this article (doi:10.1007/s10067-016-3359-x) contains supplementary material, which is available to authorized users.

## Introduction

Safety data collected during the development of new rheumatoid arthritis (RA) therapies are generally derived from randomised controlled trials (RCTs) and long-term extension (LTE) studies. Individual RCTs typically include small patient numbers and limited treatment periods [1, 2]. Control treatment duration may limit the exposure available to derive relative safety measures with concurrent internal controls, particularly for events of low frequency or long latency. Although LTE studies are conducted over longer treatment periods, many of these typically do not include a comparator arm, are open-label and may lack generalisability due to possible selection biases [2]; such factors complicate comparisons with controls.

Tofacitinib is an oral Janus kinase inhibitor for the treatment of RA. Tofacitinib clinical efficacy and safety have been demonstrated in phase (P)3 and LTE studies (Supplementary Material) [3–9], including comparisons with methotrexate [6] and adalimumab [8], and safety data up to 96 months [10]; however, active-control trials with biologic disease-modifying antirheumatic drugs (bDMARDs) are only available up to 12 months, and there is limited precision for rare events.

To further characterise tofacitinib safety, analyses were conducted to understand how the totality of accrued patient-years (pt-yrs) of tofacitinib exposure from clinical trials could inform evaluations of safety events, expanding on recently published methodology [1, 11]. These analyses were designed to estimate the exposure needed to provide 90 % probability that the lower bound of the 95 % confidence interval (CI) of selected safety events for tofacitinib was greater than external bDMARD comparator rates. The methodology used here was not intended to provide a comprehensive overview of all possible safety outcomes, but rather to be an exemplar for a generalised approach of safety contextualisation of data collected during a clinical trial programme versus other therapies.

## Materials and methods

### External comparator populations

For this analysis, the Curtis et al. methodology was applied [1]. bDMARDs were chosen for comparison with tofacitinib, and incidence rates (IRs) for AEs of interest in patients with moderate to severe RA were obtained to determine the tofacitinib exposure needed to detect a potential increased risk of the following AEs: serious infection events (SIEs), all malignancies (excluding non-melanoma skin cancer [NMSC]), NMSC, major adverse cardiovascular events (MACE), opportunistic infections (OIs), lymphoma and gastrointestinal (GI) perforations. These AEs were selected by the study team as important potential or identified risks for RA patients that were both clinically relevant and feasible for study under the methodological framework. For instance, although pregnancy safety outcomes were of interest, there were no adequately powered, well-controlled studies of pregnant women treated with tofacitinib. For the outcomes of interest, data from multiple sources (e.g. observational studies, RCTs, meta-analyses) were used and data sources were focussed on defining and optimising comparability with the tofacitinib clinical trial programme (Table 1; Supplementary Material).

**Table 1:** Pt-yrs’ exposure to tofacitinib required for the lower bound of 95 % CI to exceed background rate in external bDMARD comparator populations assuming an increase in observed rate of 1.2×, 1.5× or 2.0×

Event	External bDMARD comparator population	IR reported for external bDMARD comparator population per 100 pt-yrs^a^	Follow-up exposure (pt-yrs) to tofacitinib required to detect an assumed increased risk relative to bDMARDs with 90 % power^b^		
			1.2×	1.5×	2.0×
SIE	Systematic review/clinical trial meta-analysis [12]	Point estimate 4.90	6151	1076	311
		Lower 95 % CI 4.41	6568	1230	348
		Upper 95 % CI 5.44	5384	985	296
Malignancies (excluding NMSC)	Clinical trials meta-analysis (Pfizer Inc 2015, data on file)	Point estimate 0.95	30,945	5704	1699
		Lower 95 % CI 0.79	38,165	6872	2004
		Upper 95 % CI 1.14	25,734	4743	1413
NMSC	Published systematic review/meta-analysis of malignancies from observational studies/clinical trials [13]	0.35	84,544	15,241	4349
	Observational studies literature review (Pfizer Inc 2015, data on file)	Low 0.21	137,762	25,444	7263
		High 1.34	22,366	3933	1146
MACE	Corrona TNFi cohort (Pfizer Inc 2013, data on file)	0.54	54,684	10,084	2942
OI	Based on published long-term follow-up data of patients with active RA treated with adalimumab [14]; published literature review of infections and bDMARD therapy among patients with RA [15]	0.25^c^	118,491	21,363	6098
Lymphoma	Observational studies literature review (Pfizer Inc 2015, data on file)	Low 0.019	1,562,987	281,872	80,494
		High 0.34	87,040	15,691	4478
GI perforation	Published claims database analysis [16, 17]	0.13	228,162	39,228	11,746
		Lower 95 % CI 0.08	370,964	66,896	19,101
		Upper 95 % CI 0.19	156,011	28,129	8030
	Published RABBIT registry data^d^ [18]	High 0.066	449,723	77,326	22,080
	Observational studies literature review (Pfizer Inc 2015, data on file)	Low 0.05	593,737	107,072	30,575
		High 0.19	156,011	28,129	8030

Shaded cells highlight events that have accrued the needed tofacitinib exposure for the detection of respective 1.2×, 1.5× or 2.0×-fold increased risk, should they occur, based upon trial data current through March 2015

*bDMARD* biologic disease-modifying antirheumatic drug, *CI* confidence interval, *Corrona* Consortium of Rheumatology Researchers of North America, *EU* European, *GI* gastrointestinal, *IR* incidence rate, *MACE* major adverse cardiovascular event, *ND* not determined, *NMSC* non-melanoma skin cancer, *OI* opportunistic infection, *pt-yrs* patient years, *RA* rheumatoid arthritis, *RABBIT* Rheumatoid Arthritis Observational Biological Therapy Register, *SIE* serious infection event, *TNFi* tumour necrosis factor inhibitor

^a^Low and high refer to the lowest and highest IR values, respectively, from a range of reported values

^b^The estimated tofacitinib exposure by April 2015 within the RA clinical programme is 19,406 pt-yrs

^c^No CI available

^d^The low value was 0 (golimumab data); therefore, exposure data could not be calculated

### Tofacitinib clinical trial population

The tofacitinib clinical trial RA population included patients from two P1 studies, nine P2 RCTs, six P3 RCTs and two open-label LTE studies (Supplementary Material). One LTE study was ongoing at the time of analysis (March 2015 data-cut; unlocked).

### Analysis

Background rates of AEs were derived from external bDMARD comparator populations, as described previously. IRs were expressed as the number of unique patients with events per 100 pt-yrs’ exposure, assuming a constant hazard over time. Sample size calculations were based on a Poisson distribution to estimate the minimum pt-yrs’ exposure needed to have 90 % power to detect that the lower bound of the CI of tofacitinib/bDMARD would be >1, assuming that the true tofacitinib IRs were 1.2×, 1.5× or 2.0× greater than bDMARD IRs; multiplier thresholds were based on clinical relevance and 1.5× was used by Curtis et al., as agreed upon with the FDA for characterising tocilizumab safety [1].

Accrued exposure data collected during tofacitinib studies (P123LTE) were pooled for analysis. The number of pt-yrs’ exposure within the pooled dataset was compared with the calculated minimum tofacitinib exposure to detect a 1.2×, 1.5× or 2.0× increase in risk over external bDMARD comparator populations for each AE.

## Results

**Fig. 1:**
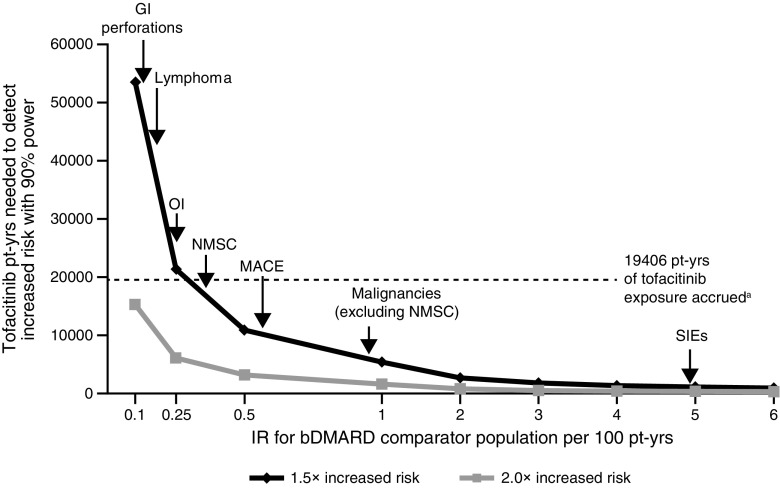
Nomogram showing the minimum amount of tofacitinib exposure (pt-yrs) required to detect a significant difference of 1.5× and 2.0× versus a bDMARD comparator in relation to example background event rates. ^a^ As of March 2015. IRs for AEs of interest are based on estimated values from the data presented in Table 1. *AE* adverse event, *bDMARD* biologic disease-modifying antirheumatic drug, *GI* gastrointestinal, *IR* incidence rate, *MACE* major adverse cardiovascular event, *NMSC* non-melanoma skin cancer, *pt-yrs* patient-years, *OI* opportunistic infection, *SIE* serious infection event

As of March 2015, 19,406 pt-yrs’ tofacitinib exposures (all doses) were accrued from 6194 patients across the P123LTE database. IRs for AEs of interest in the external bDMARD comparator populations, with similar characteristics to patients enrolled in the tofacitinib clinical trial programme, are shown in Table 1. A nomogram was developed to estimate the number of pt-yrs’ tofacitinib exposure required to detect increases in AEs of various frequencies (Fig. 1).

Based on 19,406 pt-yrs’ tofacitinib exposure, sufficient data were available to detect potential increases over estimated background bDMARD IRs in SIEs (≥1.2×), malignancies (excluding NMSC), NMSC, MACE and lymphoma (each ≥1.5), should they exist. Given the rarity of OIs and GI perforations reported in RA populations, the accrued pt-yrs’ exposure allows for the detection of a potential two-fold increase with tofacitinib relative to bDMARD IRs.

## Discussion

A robust clinical trial safety database is important for the risk/benefit assessment of a new molecular entity (NME) and should meet regulator recommendations on the extent of population exposure to assess clinical safety [19]. At the time of initial registration, RA clinical trials predominantly focussed on comparisons with placebo, with pooling across trials to achieve the necessary extent of exposure. However, as the number of effective RA therapies has increased and ethical considerations have further limited the length of placebo exposure in contemporary clinical trial programmes, interest has shifted to characterising the safety profile of new medications versus other active medications rather than placebo.

Modern development programmes generally include head-to-head or active comparator trials, with the primary intent of benchmarking the efficacy of NME. While the duration of active comparator trials is typically longer compared with placebo-controlled trials and is not as restrictive in terms of duration of exposure, logistical considerations, including sample size, study duration and event frequency, continue to limit the ability to draw precise comparisons for events with low frequency or long latency.

Furthermore, the challenge remains as to how to determine a priori how much data constitutes a sufficiently large safety database in such a clinical trial programme. Due to these difficulties, there is utility in alternative methods to compare the evidence of safety events between active therapies. Our approach, which focussed on confirmation that the minimum drug exposure within the tofacitinib clinical trial programme has been achieved to confidently ascertain whether AE rates are higher than other available therapies, may improve the utility of other clinical trial programme databases.

The data suggest that tofacitinib exposure from the clinical programme is sufficient to detect possible risk differences from bDMARDs of ≥1.2–1.5× for several AEs of interest; however, for OIs and GI perforations, only risk differences of ≥2× could be detected due to the lower frequency of these events within the available data sources. This reflects the inherent limitation of event frequency in precisely comparing such events. For such rare events, the potential risk differences that can be detected using this method may be considered insufficient to fully inform risk/benefit assessments. This limitation highlights the important role of observational studies, conducted in larger and more diverse patient populations, to assess the relative frequency of such events and those with long latency periods. Such characterisation can be achieved through prospective active surveillance within register frameworks and routine pharmacovigilance surveillance within the clinical practice setting. Ideally, one could potentially pool clinical trial data, continued by real-world observation, to provide maximal person-time follow-up.

In this analysis, limitations were introduced from the data sources used. The paucity of data available from clinical trials for some AEs of interest required the use of observational data sources and meta-analyses to provide the necessary IRs for these events in cohorts of patients receiving bDMARDs. The addition of observational sources, rather than only clinical trial data, increased the breadth of IRs available and may have influenced the sensitivity to detect a potential increased risk with tofacitinib relative to bDMARDs. These data, while valuable, introduce a wider range of patient characteristics and risk factors representative of clinical practice versus trial-specific inclusion/exclusion criteria, as well as heterogeneity in case definitions and the methods of outcome ascertainment.To address these limitations, where possible, bDMARD populations were selected to optimise similarity of comparisons with the tofacitinib clinical trial database and provide a conservative range of IRs.

A similar analytic approach was used for tocilizumab for regulatory purposes, where the number of tocilizumab-exposed pt-yrs needed to detect a ≥50 % increase in risk for key safety events versus a bDMARD population (90 % power) was determined. The overall methodologies were similar, except that within this case example, additional efforts to ‘harmonise’ the comparator patient populations were made, and all tofacitinib-related person-time was accrued within the clinical trial setting versus the combination of clinical trial and person-time estimates based on post-marketing exposure for tocilizumab [1].

The methodology described here for the tofacitinib clinical trial programme provides clinicians and experts in pharmacovigilance with an alternative way to gain perspective on the pt-yrs’ exposure required to evaluate differences in safety events of interest relative to comparator therapies. The AEs selected for this analysis were those identified by the study team as the most clinically relevant potential risks for RA patients. However, the methodology described herein could be applied to evaluate the relative risk of other safety outcomes of interest, such as abnormal laboratory test results associated with clinically relevant outcomes, which have been reported elsewhere in tofacitinib-treated RA patients [20, 21]. This approach could support the assessment of comparative safety outcomes for new RA medications. Based on our dataset, a nomogram was developed to estimate the number of pt-yrs’ tofacitinib exposure required to detect potential increases in risk versus bDMARDs, as a tool that could be more broadly applied in the design of long-term safety clinical trials and real-world safety comparisons. A 90 % power for exposure calculations was selected to ensure a higher chance of detecting potential differences rather than a lower threshold. Consequently, the tofacitinib exposure calculated represents a conservative estimate of the total pt-yrs follow-up.

Although our case study used a superiority hypothesis for safety events, non-inferiority testing would result in similar exposure sample size cutoffs for each AE of interest within non-inferiority margin set at the same values, as described for each event within the nomogram (1.5×, 2.0×). In non-inferiority testing, if the upper bound of 95 % CI based on the observed tofacitinib rate was <1.5× and 2.0× of bDMARD comparator rates, then 1.5× and 2.0× risks could be excluded. Therefore, regardless of superiority test (to detect assumed difference) or non-inferiority test (to exclude certain difference), similar inference could be drawn; an assumption of the analysis was that the IR of AEs for bDMARDs were constant over time. Whether this is a valid assumption can be tested as accumulating data allows.

The tofacitinib data are derived entirely from clinical trial data sources. Given that >55,000 patients worldwide have received tofacitinib as of May 2015 (data on file), in post-marketing experience, additional perspective for the evaluation of rare events in this more heterogeneous real-world patient and prospective active surveillance using registries is ongoing to fully define the risks associated with therapy. Future safety analyses might also include alternative comparator therapies within such registries, which would complement both the comparative clinical trials and the methods described here. Comparative safety outcome trials (NCT02092467/A3921133) are underway, but data will not be available in the near future. The creation of a safety data repository of blinded patient-level data to permit cross-comparison between existing therapies and new medications could allow for standardisation of cohort and outcome definitions and better comparability for key subgroups. This approach has been preliminarily successful in trying to harmonise international comparisons between observational RA registries [22, 23]. Additionally, a Bayesian approach, incorporating the latest safety estimates, could be considered. The current estimated sample size ignores the event rates associated with tofacitinib; this analytic framework is suitable for planning how much exposure data might be needed before actual tofacitinib rates are available.

To conclude, our risk characterisation approach represents an indirect method to contextualise the safety profile of newly introduced RA medications versus established therapies, and provides clinicians and regulatory authorities with relevant context to inform labelling and treatment choices for RA patients. To date, the safety of tofacitinib appears similar to approved published data from bDMARDs with respect to SIEs, all malignancies (excluding NMSC), NMSC, MACE, OIs, lymphoma and GI perforations.

## Electronic supplementary material

Below is the link to the electronic supplementary material.

ESM 1 . (DOC 107 kb)
