# Supplementary material for: Use of a risk characterisation approach to contextualise the safety profile of new rheumatoid arthritis treatments: a case study using tofacitinib
Source: Clin Rheumatol. 2016 Jul 28;36(3):683–8. doi: 10.1007/s10067-016-3359-x (PMC5323490; doi:10.1007/s10067-016-3359-x)
Supplement: Supplementary file 1 — . (DOC 107 kb) [file 10067_2016_3359_MOESM1_ESM.doc]

**Supplementary Material**

**Use of a risk characterisation approach to contextualise the safety profile of new rheumatoid arthritis treatments: a case study using tofacitinib**

*Clin Rheumatol*

Jeffrey R. Curtis, Richard Zhang, Sriram Krishnaswami, Andrew Anisfeld, Yan Chen, Sander Strengholt, Connie Chen, Jamie Geier **Corresponding author:** Jamie Geier, Pfizer Inc, 235 East 42nd Street, New York, NY 10017, USA; Tel: +001 212-733-8916; Email: Jamie.Geier@pfizer.com

**Methods**

**Systematic review and meta-analysis of published interventional studies**

The systematic review and meta-analysis of published interventional studies with biologic disease-modifying antirheumatic drugs (bDMARDs) in rheumatoid arthritis (RA) were conducted to estimate incidence rates (IRs; unique patients with events per 100 pt-yrs’ exposure) for specific adverse events (AEs) with bDMARDs. Systematic review of the literature involved synthesis of published randomised controlled trial (RCT) and long-term extension (LTE) data in adult patients with moderate‑to‑severe RA. One meta-analysis analysed data for serious infection events (SIEs) [1], a separate meta-analysis was performed for all malignancies (excluding non‑melanoma skin cancer [NMSC]) (Pfizer Inc 2015, data on file). Preferred Reporting Items for Systematic reviews and Meta-Analyses (PRISMA) consensus was followed, with each literature search conducted according to the Participants, Interventions, Comparisons, Outcome, Study Design (PICOS) statement [2]. Interventions included biologic agents currently licensed by the US Food and Drug Administration and/or European Medicines Agency for the treatment of RA. Articles published in English, in Medline, Embase, and Biosis, through October 2013 (SIEs) or August 2014 (malignancies [excluding NMSC]) were selected. Regulatory submissions within the US Food and Drug Administration Summary Basis for Approvals, as well as European Public Assessment Reports, were searched for data for bDMARDs approved for moderate‑to‑severe RA. Data were selected to match the population of tofacitinib trials: adults with moderate‑to‑severe, active RA. Trials were required to report data for the AE of interest and have sufficient data to calculate IRs (i.e., number of patients and patient-years [pt-yrs] of exposure). Study designs included RCTs and LTE studies. Studies that combined two biologic agents, duplicate reports, economic assessments, editorial comments, and case reports were excluded.

Search criteria included keywords such as ‘rheumatoid arthritis’ and clinical trials of bDMARDs (abatacept, adalimumab, certolizumab, etanercept, golimumab, infliximab, rituximab, and tocilizumab). Meta-analysis of the data extracted from the studies that met selection criteria was conducted to summarise and contextualise the risk of SIEs or malignancies (excluding NMSC) with bDMARDs. Point estimates for IRs (95% confidence interval [CI]) across bDMARDs were estimated based on RCT and LTE data using a random effects meta-analytic model with a restricted maximum likelihood estimator for between-study variances.

*Literature review of published observational studies*

A literature review of published observational studies involving patients with adult-onset RA was conducted to evaluate IRs for NMSC, lymphoma and gastrointestinal (GI) perforations for bDMARDs (Pfizer Inc 2015, data on file). Articles published in English, in PubMed, through September 2015 were selected. Studies were required to report IR data for the AE of interest or have sufficient data to calculate IRs. Study designs included observational studies. Studies that reported exclusively on clinical trial participants, literature reviews or meta-analyses, and case reports, were excluded.

Search criteria included keywords such as ‘rheumatoid arthritis’, ‘incidence’ and search terms for malignancies and GI perforations. Sub-analysis of data identified for malignancies was performed for NSMC and lymphoma. IRs (unique patients with events per 100 pt-yrs) were calculated from raw data if IRs were not reported in each publication for bDMARDs and crude IRs were reported.

*US registry*

Analysis of AE data (cut-off: March 2013) from patients treated with tumour necrosis factor inhibitors included in the Consortium of Rheumatology Researchers of North America (Corrona) registry (similar disease severity and demographics to the tofacitinib clinical trial programme) provided the IR for MACE (Corrona 2013, data on file).

*Individual published sources of safety information*

Individual published sources of safety information for bDMARD-treated patients were included for NMSC, OIs, lymphoma and GI perforation, given either the rarity and/or lack of published comparative data for these events. These sources included: for NMSC, a published systematic review and meta-analysis of malignancies from registries and prospective observational studies [3]; for OIs, a literature review of infections and bDMARD therapy among patients with RA [4] and long-term follow-up data of patients with active RA treated with adalimumab and methotrexate [5] and for GI perforation, data from patients treated with TNFi within a claims database as reported for contextualisation purposes within a summary report for tocilizumab [6] and analysis of lower GI perforation events from the Rheumatoid Arthritis Observational Biological Therapy Register (RABBIT) registry (2001–April 2015) for bDMARDs (range of IRs reported) [7].

**Tofacitinib clinical trial population**

The tofacitinib clinical trial RA population included in the analysis involved patients with active RA treated in P1, P2, and P3 studies, and open-label LTE studies (Table 1); approximately 14,900 pt-yrs’ exposure were derived from LTE studies.

**Supplementary Table**

**Table 1.** Summary of the P1, P2, P3 and LTE studies of tofacitinib in RA included in analysis of tofacitinib exposure (cut-off: March 2015)

| **Study** | **Tofacitinib doses and RA patient population** | **N**  **(Patients randomised and who received ≥1 dose of study drug)** | **Control group** | **Treatment duration** |
| --- | --- | --- | --- | --- |
| **P1 studies** | | | | |
| NCT01262118/ A3921130  [8] | 10 mg BID with or without background MTX in pts on stable MTX (for MTX treated) with active RA | 36 | – | 6 wk |
| NCT01484561/ A3921152  [9] | 10 mg BID in pts with an IR to at least one cs- or bDMARD due to lack of efficacy or intolerance. Pts on stable csDMARDs remained on this treatment | 148 | PBO | 6 wk |
| **P2 studies** | | | | |
| NCT00147498/ A3921019  [10] | 5, 15, or 30 mg BID (monotherapy) in pts with an IR to MTX, etanercept, infliximab, or adalimumab | 264 | PBO | 6 wk |
| NCT00413660/ A3921025 [11] | 1, 3, 5, 10, or 15 mg BID or 20 mg QD in MTX-IR patients receiving background MTX | 507 | PBO | 24 wk |
| NCT00550446/ A3921035  [12] | 1, 3, 5, 10, or 15 mg BID (monotherapy) in DMARD-IR pts | 384 | PBO or adalimumab 40 mg every 2 weeks | 24 wk |
| NCT00603512/ A3921039  [13] | 1, 3, 5, or 10 mg BID in Japanese MTX-IR pts receiving background MTX | 136 | PBO | 12 wk |
| NCT00687193/ A3921040 [14] | 1, 3, 5, 10, or 15 mg BID (monotherapy) in Japanese DMARD-IR pts | 317 | PBO | 12 wk |
| NCT01164579/ A3921068 [15] | 10 mg BID + MTX (combination therapy), tofacitinib 10 mg BID (monotherapy) in MTX naïve pts | 109 | MTX, PBO | 12 mo |
| NCT00976599/ A3921073 [16] | 10 mg BID in MTX-IR pts on background MTX | 29 | PBO | 28 days |
| NCT01059864/ A3921109 [17] | 10 mg BID (monotherapy) for 6 wks, randomized to PBO or atorvastatin for a further 6 wks in pts with active RA | 111 | PBO (+ tofacitinib) versus atorvastatin (+ tofacitinib) | 6 wks plus  6 wks |
| NCT01359150/ A3921129  [18] | 10 mg BID with and without background MTX | 200 | PBO | 64 days |
| **P3 studies** | | | | |
| ORAL Step/ NCT00960440/ A3921032 [19] | 5 or 10 mg BID in TNFi-IR pts on background MTX | 399 | PBO (adv to tofacitinib at Mo 3) | 6 mo |
| ORAL Scan/ NCT00847613/ A3921044 [20] | 5 or 10 mg BID in MTX-IR pts on background MTX | 797 | PBO (adv to tofacitinib at Mo 3 or 6) | 24 mo |
| ORAL Solo/ NCT00814307/ A3921045 [21] | 5 or 10 mg BID (monotherapy) in cs- or bDMARD-IR pts | 610 | PBO (adv to tofacitinib at Mo 3) | 6 mo |
| ORAL Sync/ NCT00856544/ A3921046 [22] | 5 or 10 mg BID in cs- or bDMARD-IR pts on background csDMARDs | 792 | PBO (adv to tofacitinib at Mo 3 or 6) | 12 mo |
| ORAL Standard/ NCT00853385/ A3921064 [23] | 5 or 10 mg BID in MTX-IR pts on background MTX | 717 | PBO or adalimumab 40 mg every 2 weeks  (PBO adv to tofacitinib at Mo 3 or 6) | 12 mo |
| ORAL Start/ NCT01039688/ A3921069 [24] | 5 or 10 mg BID (monotherapy) in MTX naïve pts | 956 | MTX | 24 mo |
| **LTE studies** | | | | |
| ORAL Sequel/ NCT00413699/ A3921024 (ongoing) [25] | 5 or 10 mg BID^a^ in pts from qualifying tofacitinib index studies, as monotherapy or with background csDMARDs | Ongoing | – | As required |
| NCT00661661/ A3921041 [25] [26] | 5 or 10 mg BID^a,b^ in Japanese pts from qualifying tofacitinib index studies, as monotherapy or with background csDMARDs | 486 | – | As required |

^a^ Dosage could be adjusted between tofacitinib 5 mg BID and tofacitinib 10 mg BID doses
^b^ Although doses are listed as tofacitinib 5 mg BID and tofacitinib 10 mg BID for this study, all patients initiated treatment at tofacitinib 5 mg BID

Adv, advanced; BID, twice daily; bDMARD, biologic disease-modifying antirheumatic drug; csDMARD, conventional synthetic disease-modifying antirheumatic drug; DMARD, disease-modifying antirheumatic drug; IR, inadequate response; LTE, long-term extension; mo, month; MTX, methotrexate; P, phase; PBO, placebo; pts, patients; QD, once daily; LTE, long-term extension; MTX, methotrexate; RA, rheumatoid arthritis; TNFi, tumour necrosis factor inhibitor; wk, week

**References**

1. Strand V, Ahadieh S, French J, Geier J, Krishnaswami K, Menon S, Checchio T, Tensfeldt T, Hoffman E, Riese R, Boy M, Gomez-Reino J (2015) Systematic review and meta-analysis of serious infections with tofacitinib and biologic disease-modifying antirheumatic drug treatment in rheumatoid arthritis clinical trials. Arthritis Res Ther 17:1–9.

2. Liberati A, Altman DG, Tetzlaff J, Mulrow C, Gotzsche PC, Ioannidis JP, Clarke M, Devereaux PJ, Kleijnen J, Moher D (2009) The PRISMA statement for reporting systematic reviews and meta-analyses of studies that evaluate healthcare interventions: explanation and elaboration. BMJ 339:b2700

3. Mariette X, Matucci-Cerinic M, Pavelka K, Taylor P, van Vollenhoven R, Heatley R, Walsh C, Lawson R, Reynolds A, Emery P (2011) Malignancies associated with tumour necrosis factor inhibitors in registries and prospective observational studies: a systematic review and meta-analysis. Ann Rheum Dis 70:1895–1904.

4. Winthrop KL (2012) Infections and biologic therapy in rheumatoid arthritis: our changing understanding of risk and prevention. Rheum Dis Clin North Am 38:727–745.

5. Keystone EC, van der Heijde D, Kavanaugh A, Kupper H, Liu S, Guérette B, Mozaffarian N (2013) Clinical, functional, and radiographic benefits of longterm adalimumab plus methotrexate: final 10-year data in longstanding rheumatoid arthritis. Journal of Rheumatology 40:1487–1497.

6. Gout T, Ostor AJ, Nisar MK (2011) Lower gastrointestinal perforation in rheumatoid arthritis patients treated with conventional DMARDs or tocilizumab: a systematic literature review. Clin Rheumatol 30:1471–1474.

7. Strangfeld A, Richter A, Herzer P, Rockwitz K, Demary W, Aringer M, Zink A, Listing J (2015) Risk for lower intestinal perforations in RA patients treated with tocilizumab in comparison to treatment with TNF inhibitors, rituximab, abatacept or conventional synthetic DMARDs. Arthritis Rheum 67:

8. Charles-Schoeman C, Fleischmann R, Davignon J, Schwartz H, Turner SM, Beysen C, Milad M, Hellerstein MK, Luo Z, Kaplan IV, Riese R, Zuckerman A, McInnes IB (2015) Potential mechanisms leading to the abnormal lipid profile in patients with rheumatoid arthritis versus healthy volunteers and reversal by tofacitinib. Arthritis Rheumatol 67:616–625.

9. Kremer JM, Kivitz AJ, Simon-Campos JA, Nasonov EL, Tony HP, Lee SK, Vlahos B, Hammond C, Bukowski J, Li H, Schulman SL, Raber S, Zuckerman A, Isaacs JD (2015) Evaluation of the effect of tofacitinib on measured glomerular filtration rate in patients with active rheumatoid arthritis: results from a randomised controlled trial. Arthritis Res Ther 17:95

10. Kremer JM, Bloom BJ, Breedveld FC, Coombs JH, Fletcher MP, Gruben D, Krishnaswami S, Burgos-Vargas R, Wilkinson B, Zerbini CAF, Zwillich SH (2009) The safety and efficacy of a JAK inhibitor in patients with active rheumatoid arthritis: Results of a double-blind, placebo-controlled phase IIa trial of three dosage levels of CP-690,550 versus placebo. Arthritis Rheum 60:1895–1905.

11. Kremer JM, Cohen S, Wilkinson BE, Connell CA, French JL, Gomez-Reino J, Gruben D, Kanik KS, Krishnaswami S, Pascual-Ramos V, Wallenstein G, Zwillich SH (2012) A phase IIb dose-ranging study of the oral JAK inhibitor tofacitinib (CP-690,550) versus placebo in combination with background methotrexate in patients with active rheumatoid arthritis and an inadequate response to methotrexate alone. Arthritis Rheum 64:970–981.

12. Fleischmann R, Cutolo M, Genovese MC, Lee EB, Kanik KS, Sadis S, Connell CA, Gruben D, Krishnaswami S, Wallenstein G, Wilkinson BE, Zwillich SH (2012) Phase IIb dose-ranging study of the oral JAK inhibitor tofacitinib (CP-690,550) or adalimumab monotherapy versus placebo in patients with active rheumatoid arthritis with an inadequate response to disease-modifying antirheumatic drugs. Arthritis Rheum 64:617–629.

13. Tanaka Y, Suzuki M, Nakamura H, Toyoizumi S, Zwillich SH, Tofacitinib Study Investigators (2011) Phase II study of tofacitinib (CP-690,550) combined with methotrexate in patients with rheumatoid arthritis and an inadequate response to methotrexate. Arthritis Care Res (Hoboken) 63:1150–1158.

14. Tanaka Y, Takeuchi T, Yamanaka H, Nakamura H, Toyoizumi S, Zwillich S (2015) Efficacy and safety of tofacitinib as monotherapy in Japanese patients with active rheumatoid arthritis: a 12-week, randomized, phase 2 study. Mod Rheumatol 25:514–521.

15. Conaghan PG, Ostergaard M, Bowes MA, Wu C, Fuerst T, van der Heijde D, Irazoque-Palazuelos F, Soto-Raices O, Hrycaj P, Xie Z, Zhang R, Wyman BT, Bradley JD, Soma K, Wilkinson B (2016) Comparing the effects of tofacitinib, methotrexate and the combination, on bone marrow oedema, synovitis and bone erosion in methotrexate-naive, early active rheumatoid arthritis: results of an exploratory randomised MRI study incorporating semiquantitative and quantitative techniques. Ann Rheum Dis [Epub ahead of print]:

16. Boyle DL, Soma K, Hodge J, Kavanaugh A, Mandel D, Mease P, Shurmur R, Singhal AK, Wei N, Rosengren S, Kaplan I, Krishnaswami S, Luo Z, Bradley J, Firestein GS (2015) The JAK inhibitor tofacitinib suppresses synovial JAK1-STAT signalling in rheumatoid arthritis. Ann Rheum Dis 74:1311–1316. doi: Study 1073

17. McInnes IB, Kim HY, Lee SH, Mandel D, Song YW, Connell CA, Luo Z, Brosnan MJ, Zuckerman A, Zwillich SH, Bradley JD (2014) Open-label tofacitinib and double-blind atorvastatin in rheumatoid arthritis patients: a randomised study. Ann Rheum Dis 73:124–131.

18. Winthrop KL, Silverfield J, Racewicz A, Neal J, Lee EB, Hrycaj P, Gomez-Reino J, Soma K, Mebus C, Wilkinson B, Hodge J, Fan H, Wang T, Bingham CO, III (2016) The effect of tofacitinib on pneumococcal and influenza vaccine responses in rheumatoid arthritis. Ann Rheum Dis 75:687–695.

19. Burmester GR, Blanco R, Charles-Schoeman C, Wollenhaupt J, Zerbini C, Benda B, Gruben D, Wallenstein G, Krishnaswami S, Zwillich SH, Koncz T, Soma K, Bradley J, Mebus C, ORAL Step investigators (2013) Tofacitinib (CP-690,550) in combination with methotrexate in patients with active rheumatoid arthritis with an inadequate response to tumour necrosis factor inhibitors: a randomised phase 3 trial. Lancet 381:451–460.

20. van der Heijde D, Tanaka Y, Fleischmann R, Keystone E, Kremer J, Zerbini C, Cardiel MH, Cohen S, Nash P, Song YW, Tegzová D, Wyman BT, Gruben D, Benda B, Wallenstein G, Krishnaswami S, Zwillich SH, Bradley JD, Connell CA, ORAL Scan Investigators (2013) Tofacitinib (CP-690,550) in patients with rheumatoid arthritis receiving methotrexate: twelve-month data from a twenty-four-month phase III randomized radiographic study. Arthritis Rheum 65:559–570.

21. Fleischmann R, Kremer J, Cush J, Schulze-Koops H, Connell CA, Bradley JD, Gruben D, Wallenstein GV, Zwillich SH, Kanik KS, ORAL Solo Investigators (2012) Placebo-controlled trial of tofacitinib monotherapy in rheumatoid arthritis. N Engl J Med 367:495–507.

22. Kremer J, Li ZG, Hall S, Fleischmann R, Genovese M, Martin-Mola E, Isaacs JD, Gruben D, Wallenstein G, Krishnaswami S, Zwillich SH, Koncz T, Riese R, Bradley J (2013) Tofacitinib in combination with nonbiologic disease-modifying antirheumatic drugs in patients with active rheumatoid arthritis: a randomized trial. Ann Intern Med 159:253–261.

23. van Vollenhoven RF, Fleischmann R, Cohen S, Lee EB, García Meijide JA, Wagner S, Forejtova S, Zwillich SH, Gruben D, Koncz T, Wallenstein GV, Krishnaswami S, Bradley JD, Wilkinson B, ORAL Standard Investigators (2012) Tofacitinib or adalimumab versus placebo in rheumatoid arthritis. N Engl J Med 367:508–519.

24. Lee EB, Fleischmann R, Hall S, Wilkinson B, Bradley J, Gruben D, Koncz T, Krishnaswami S, Wallenstein G, Zang C, Zwillich S, van Vollenhoven R, on behalf of the ORAL Start investigators (2014) Tofacitinib versus methotrexate in rheumatoid arthritis. N Engl J Med 370:2377–2386.

25. Wollenhaupt J, Silverfield J, Lee EB, Curtis JR, Wood SP, Soma K, Nduaka CI, Benda B, Gruben D, Nakamura H, Komuro Y, Zwillich SH, Wang L, Riese RJ (2014) Safety and efficacy of tofacitinib, an oral Janus kinase Inhibitor, for the treatment of rheumatoid arthritis in open-label, longterm extension studies. J Rheumatol 41:837–852. doi: 53420

26. Yamanaka H, Tanaka Y, Takeuchi T, Sugiyama N, Yuasa H, Toyoizumi S, Morishima Y, Hirose T, Zwillich SH (2016) Tofacitinib, an oral Janus kinase inhibitor, as monotherapy or with background methotrexate, in Japanese patients with rheumatoid arthritis: an open-label, long-term extension study. Arthritis Res Ther 18:34
